# The legacy of the COVID-19 pandemic for the healthcare environment: the establishment of long COVID/ Post-COVID-19 condition follow-up outpatient clinics in Germany

**DOI:** 10.1186/s12913-025-12521-2

**Published:** 2025-03-10

**Authors:** Lucas C. Adam, Fabian Boesl, Vanessa Raeder, Ameli Breuer, Benno Bremer, Heinrich J. Audebert, Christiana Franke

**Affiliations:** Department of Neurology and Experimental Neurology, Charité–Universitätsmedizin Berlin, Corporate Member of Freie Universität Berlin and Humboldt-Universität Zu Berlin, Hindenburgdamm 30, Berlin, 12203 Germany

**Keywords:** Post-COVID-19 syndrome (PCS), Post-COVID-19 condition (PCC), Long-COVID, Outpatient clinic, Follow-up care, Post-acute sequelae of COVID-19 (PASC), Post-acute infection syndrome (PAIS)

## Abstract

**Background:**

Since 2020, several specialized follow-up outpatient clinics have been established across Germany to address the complex needs of patients with Long COVID/ Post-COVID-19 Condition (PCC). This article reviews the current landscape of these specialized clinics in Germany and critically evaluates their diagnostic and treatment algorithms.

**Methods:**

This study employed a mixed-method approach, combining publicly available information on post-COVID-19 outpatient clinics with an observational cross-sectional online survey among lead doctors of PCC follow-up outpatient clinics in Germany. The survey was conducted from November 2023 to January 2024. Descriptive statistics and t-tests for group-comparisons were employed, with statistical significance set at *p* < 0.05.

**Results:**

At the time of the survey, 112 specialized PCC outpatient clinics were identified in Germany through publicly available information. Forty-five PCC outpatient clinic lead doctors (40.2%) responded to our survey. Treatment of PCC patients is personalized and symptom-oriented rather than standardized. Patient characteristics of the two identified main treatment domains, focusing on respiratory and neurocognitive symptoms, differed only in sex distribution. A higher proportion of females (63.9%) presented with pulmonary symptoms compared to patients with neurocognitive impairments (50.2%, *p* < 0.05). The level of distress among patients is generally perceived as high and outpatient clinic lead doctors are convinced that their outpatient counseling services offer significant benefits.

**Conclusions:**

As the demand for PCC follow-up outpatient clinics persists, the establishment of new services continues, particularly to address the growing need for neurocognitive care services. PCC outpatient care is currently personalized and symptom-orientated, leading to high variability across clinics. Further standardization of treatment protocols and diagnostic algorithms could improve patient care and facilitate professional exchange.

**Supplementary Information:**

The online version contains supplementary material available at 10.1186/s12913-025-12521-2.

## Background

The sequelae of the SARS-CoV-2 pandemic have led to a multifaceted and socioeconomic predicament, manifesting as long-term health effects known as Post-Acute Sequelae of COVID-19 (PASC), Long/Post COVID, Post-COVID-19 Syndrome (PCS), Post-COVID-19 Condition (PCC). According to the Delphi consensus statement, PCC is characterized by persistent or newly emerging symptoms lasting for at least three months from the onset of COVID-19, which cannot be attributed to another condition [1]. With more than 65 million cases worldwide, PCC is the pandemic after the pandemic, posing a substantial health and economic burden [2]. Up to 5% of all COVID-19 survivors develop at least one PCC symptom between 6 and 18 months after SARS-CoV-2 infection [3]. Symptoms range from debilitating pulmonary symptoms to stress-dependent neurocognitive impairments, such as memory, concentration, and attention deficits. Risk factors associated with PCC development include female sex, low socioeconomic status, membership in minority groups, and pre-existing conditions such as type 2 diabetes, asthma, or psychiatric conditions such as anxiety disorders, depression and post-traumatic stress disorder [4, 5]. Many PCC patients experience limitations in their daily functioning, impacting social participation and quality of life [6–8].

To date, the underlying pathomechanisms remain unclear, although previous reports suggest a complex interplay of post-viral immune dysregulation [9], persistent inflammation [10], autoimmunity [11], as well as altered stress hormone homeostasis [12]. Aside from somatic causes, some researchers suggest a predominantly psychosomatic origin contributing to PCC symptoms [13]. With no causal therapy available, current management primarily focuses on symptom-orientated therapy, preferably in a specialized setting [14].

Given the debilitating physical and psychological strain, socio-economic burden, and the complexity of heterogeneous symptoms in PCC patients, clinical treatment by a multidisciplinary team appears necessary. Specialized interdisciplinary follow-up outpatient PCC clinics can play a crucial role in coordinating patients with diverse symptomatic manifestations [15]. The specific structure and therapeutic pathways depend significantly on individual patient needs and the clinical experiences of the treating institution [14, 16, 17]. This article provides an overview of the current landscape of the PCC follow-up outpatient clinics in Germany and presents the findings from a survey conducted among the lead doctors of these clinics focusing on both the formal and substantive structure of their clinics.

## Methods

To gain a broader perspective on the current state of PCC outpatient clinics, we employed a convergent mixed-methods design that combined two distinct approaches: Publicly available data on the formal characteristics of PCC follow-up outpatient clinics were collected and linked to a primarily quantitative survey of PCC outpatient clinic lead doctors. This convergent approach leveraged the strengths of both quantitative and observational research methods, providing an innovative approach to address contemporary challenges in healthcare services [18].

### Overview of outpatient clinics

**Fig. 1:**
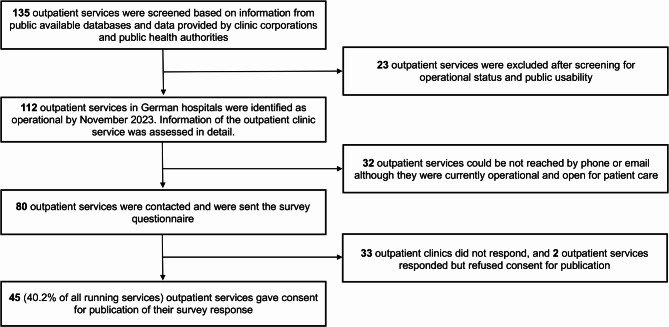
PCC outpatient clinic selection flowchart: Data from public databases and health authorities

In the absence of a centralized, up-to-date information platform in Germany regarding the procedures of follow-up PCC clinics, data was gathered from various public sources. As illustrated in Fig. 1, the initial screening of all available information on outpatient clinics involved compiling data from several sources from November 2023 to January 2024. This included a list provided by the German PCC patient group (“Long COVID Deutschland”, “https://longcoviddeutschland.org/ambulanzen/”), a platform administered by the German Federal Ministry of Health (“BMG Initiative Long COVID”, “https://www.bmg-longcovid.de/”), the information websites of federal German states (e.g., in the State of Hesse, “https://soziales.hessen.de/corona/long-covid-ambulanzen”), email contacts of the 30 largest hospital corporations in Germany. In addition, online research was conducted using publicly available search engines with the following search terms: “Post-COVID19”, “Long COVID“, “Ambulanz”, “Sprechstunde”, “[the name of one of the 16 German federal states]”. After a thorough screening procedure, 135 PCC follow-up outpatient clinics were initially identified. However, 23 clinics were excluded from further analysis due to various factors, including no longer being operational, not yet being operational at the time of the study, or having restricted accessibility—such as exclusively accepting in-house transfers, only selecting subgroups based on study inclusion criteria and not admitting public patients. After screening, each of the 112 operational outpatient clinic was contacted to verify its operational status, affiliation with a hospital, medical specialization, and the potential participation of its staff in a survey questionnaire.

### Study design and participants

The participants in the employed survey were physicians holding a leadership position within the follow-up outpatient clinic or serve as delegates of the lead physician. Each institution could participate in the survey only once. They were responsible for and familiar with the organizational structures of the outpatient clinic. To maintain the scope of a single paper, only outpatient clinics treating adults as well as being affiliated with hospitals were considered, while outpatient clinics for children or adolescents, ambulatory centers, or individual doctors’ offices were excluded. Participation in the survey was voluntary, and participants were not required to provide written consent. In accordance with Paragraph 15, Subsection 1 of the Professional Code of Conduct of the Berlin Medical Association, ethics approval was not required for this anonymized and non-personal data therefore an ethics approval was not obtained. The survey documentation included a section on participants’ consent for publication, enabling them to express their agreement to study participation. Participants were assured that their non-personalized data would be treated with strict anonymity and in accordance with the relevant guidelines and regulations.

### Data collection

Participants responded to the survey questionnaire in German from November 2023 to January 2024. The full untranslated German questionnaire, as well as an English translation, is appended as Supplement 1. We were able to contact 80 out of 112 operating outpatient clinics where public contact information by email or phone was available. The remaining 32 out of 112 clinics either failed to provide contact information, listed only functional email addresses without responding to inquiries, or had staff who were either no longer in charge or unavailable. Forty-seven clinics responded to our survey, with 45 (40.2%) providing informed consent for publication, as depicted in Fig. 1. Based on our experience with PCC patients in an outpatient clinic setting [14], a survey instrument with five distinct sections was developed: (1) formal aspects, (2) patient care organization, (3) patient perspectives, (4) treatment modalities, and (5) improvements in healthcare structure facilitated by outpatient clinics. Table 1 summarizes the main sections of the survey. Before answering the survey, participants were asked to consent to the publication of their responses.

**Table 1:** Summary of survey sections and investigated aspects

Survey sections	Investigated aspects
1. Formal structure	• Formal parameters of the outpatient clinic
	• Staffing and collaborations
	• Treatment and referral mechanisms
2. Patient care organization	• Staff - patient interaction analysis
	• Patient demographics
3. Patient perspectives	• Patient symptoms
	• Functional levels
	• Outpatient clinic enrollment expectations
4. Treatment modalities	• Diagnostic protocols
	• Treatment measures
	• Evaluation of significance of the outpatient clinics
5. Improvement in healthcare structure facilitated by outpatient clinics	• Optimization and recommendations

### Survey sections

#### Formal structure

The initial segment of the survey explored formal parameters, encompassing the commencement date and service region delineated by the outpatient clinic’s postal code. Additionally, participants were required to state the primary medical specialization of the outpatient clinic and specify upon the formal guidelines employed for PCC patient care. Subsequent items concerned the quantitative and occupational composition of the outpatient clinic’s personnel. Further inquiries addressed the collaborations between the outpatient clinics, adjacent rehabilitation facilities, and ambulatory general practitioner offices. Another item investigated the presence of educational initiatives for the outpatient clinic staff. Finally, participants were asked to elaborate on patient referral mechanisms and define the spectrum of managed disorders, including those related to the post-acute sequelae of SARS-CoV-2 infection, such as “Post/Long-COVID-19 Condition”, “post-COVID-19 vaccination syndrome”, and other related disorders.

#### Patient care organization

The second section comprised an analysis of the extent and duration of patient interactions within the outpatient clinic. The assessment included items that evaluated the number of previously enrolled patients and ascertained whether a confirmed infection, determined through polymerase chain reaction (PCR) or antigen rapid test, was a requirement for presentation. Additional metrics encompassed demographic information, including the estimated sex distribution and average age of the patient cohort.

#### Patient perspectives

The third section aimed to delineate the suffering experienced by PCC patients in outpatient clinics, including the manifestation and intensity of their symptoms. Additionally, items in this section assessed patients’ average functional levels in their daily routines and explored their expectations regarding enrollment in the outpatient clinic.

#### Treatment modalities

This section explored the diagnostic and treatment protocols of the follow-up outpatient clinics. Multiple-choice items allowed the participants to report specific diagnostic and therapeutic measures in patient care, including cognitive tests and specialized diagnostic procedures such as lumbar punctures in neurology or lung function tests in pulmonology. Lead doctors estimated the frequency of PCC diagnoses relative to the number of presenting patients and identified potential alternative diagnoses. Finally, respondents were asked about routine patient screenings for possible study enrollment and to rate the significance of the outpatient clinic in facilitating patients’ recovery.

#### Improvement in healthcare structure facilitated by outpatient clinics

The concluding section comprised a single open-response item, asking lead doctors to provide recommendations for further optimization of patient care within PCC follow-up outpatient clinics.

### Statistical analysis

Descriptive statistical analyses were performed using IBM SPSS Statistics (Version 29), encompassing calculations of frequencies, means, and medians for key variables under investigation. To accurately represent patient demographics, variables capturing general patient information, such as sex distribution, were weighted according to the number of treated patients in each outpatient clinic. Follow-up outpatient services were divided into two subgroups based on the symptom domain of treatment: (1) Respiratory-dominant symptoms and (2) Neurocognitively-dominant symptoms. Comparative subgroup analyses of outpatient clinics focusing on neurocognitive and respiratory domains were conducted using two-tailed independent t-tests to evaluate differences in formal and procedural characteristics. Statistical significance was set at *p* < 0.05.

## Results

### Public data gathering

**Fig. 2:**
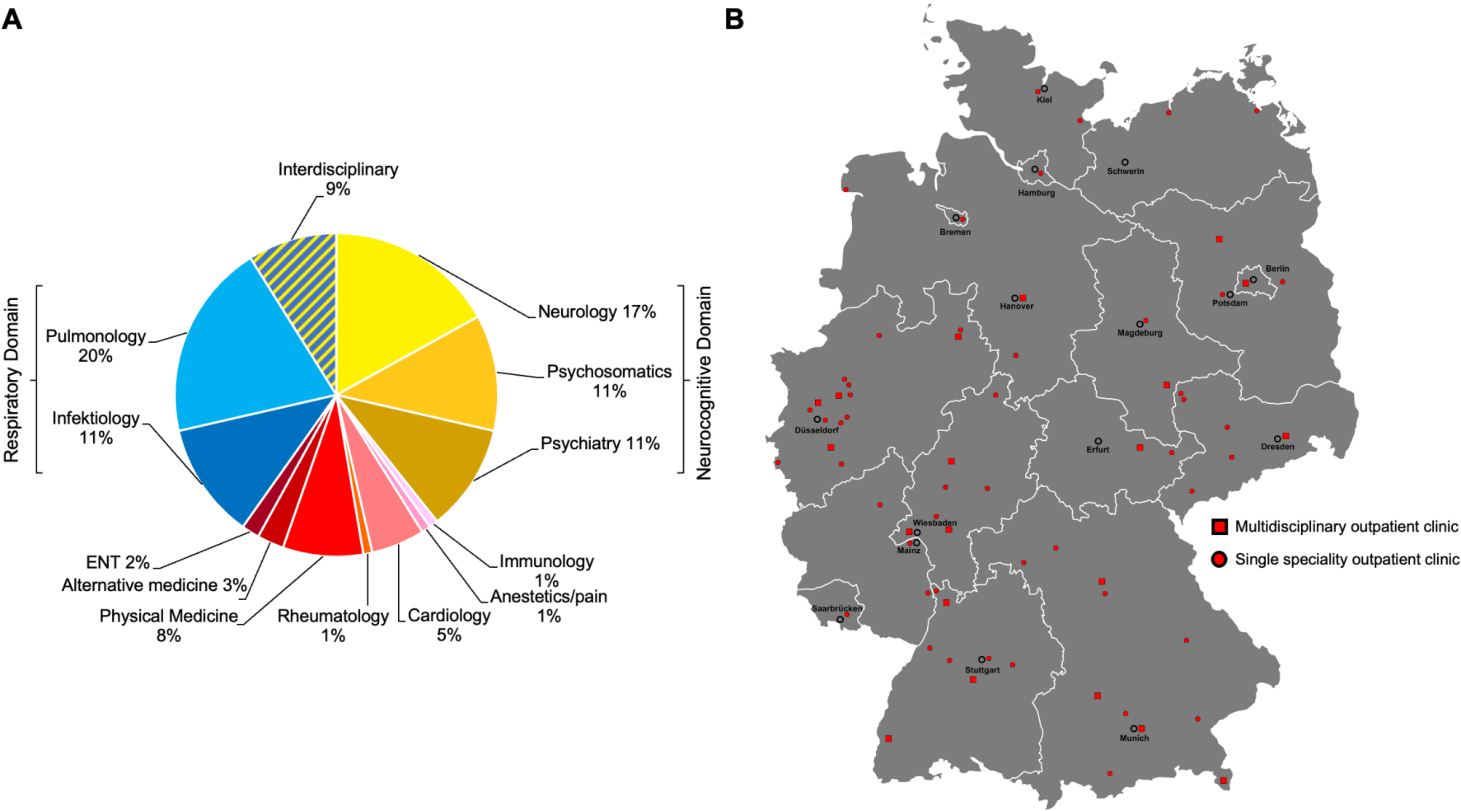
All 112 identified PCC follow-up outpatient clinics in Germany, stratified by medical specialty (**A**) and location (**B**). Red squares with black line indicate an interdisciplinary outpatient service with at least one service from the respiratory and one service from the neurocognitive domain. Red circles with black line indicate outpatient services where only one of the above-mentioned domains is covered

By January 2024, 112 operating outpatient services specialized in PCC were identified in Germany. The majority of follow-up outpatient services (91.9%) were available to all patients experiencing PCC symptoms. However, some outpatient services targeted specific patient groups. For instance, the German Bundeswehr (Armed forces of the Federal Republic of Germany) provided one (0.9%) outpatient service exclusively for its personnel at its Berlin location. Additionally, several clinics affiliated with accident insurance carriers (“Berufsgenossenschaften”) exclusively treated cases related to occupational diseases (8.0%). Most consultation services were concentrated in urban areas (85.7%) with limited availability in rural regions (Fig. 2B). Notably, most outpatient clinics were affiliated with university clinics and research facilities (57.1%). Stratification by medical discipline indicates two main areas of specialization: a respiratory domain (35 services, 31.3%), which includes pulmonary-led (19.6%) and infectiology-led (11.6%) services, and a neurocognitive domain (44 services, 39.3%), which includes outpatient clinics led by psychiatry (10.7%), neurology (16.9%) and psychosomatic departments (11.6%, Fig. 2A). The remaining services were run as interdisciplinary clinics (9%), and clinics specialized in physical medicine and rehabilitation (8.0%), cardiology (5.4%), alternative medicine (2.7%), ear, nose, and throat (ENT, 1.8%), pain/anesthetics (0.9%), rheumatology (0.9%), and immunology (0.9%).

### Survey results

#### Formal structure

According to our survey data, the first PCC outpatient clinics in Germany opened in May 2020 with the most recent one starting operations in December 2023. Among the respondents, 15.6% reported running an interdisciplinary clinic, whereas the remaining clinics have a leading medical specialty (84.4%). Participants allocated themselves to either the respiratory domain (14 responses, 31.1%), the neurocognitive domain (14 responses, 31.1%), or another medical specialty (10 responses, 22.2%), as detailed in Table 2 for outpatient clinic characteristics.

**Table 2:** Summary of key characteristics of 45 German PCC follow-up outpatient clinics that responded to the survey

Characteristics	Measure
Date of establishment (date, range)	07/2021 (05/2020–12/2023)
Total PCC patients per clinic (M, SD)	484 (543)
Leading medical specialty	
- Pulmonology (%)	10 (22%)
- Psychiatry (%)	5 (11.1%)
- Psychosomatics (%)	5 (11.1%)
- Physical Medicine (%)	5 (11.1%)
- Neurology (%)	4 (8.9%)
- Infectiology (%)	4 (8.9%)
- Other (%)	5 (11.1%)
Interdisciplinary leadership	7 (15.6%)
Admission by	
- patient himself	51.5%
- general practitioner	26.7%
- transfer from others clinics	4.4%
- internal transfer (from other disciplines	6.7%
Types of disorders treated	
- PCC	100%
- Post-Vaccine-Syndrome	40%
- Other diseases	8.9%
Screening for study involvement	66.7%

*M* Mean, *SD* Standard deviation, *PCC* Post-COVID-19 Condition

40% of respondents treated patients with post-COVID-19 vaccination sequelae, while 8.9% reported managing patients with other conditions in the PCC outpatient setting, including post-intensive care syndrome (PICS), myalgic encephalomyelitis/chronic fatigue syndrome (ME/CFS), and psychiatric disorders.

Patient care was provided according to the guidelines of AWMF S1-Leitlinie Long/Post-COVID [19] (77.8%), related publications (55.6%), personal clinical experience (55.6%), already established outpatient concepts for other disorders like ME/CFS (26.7%), and “DGN Leitlinie Neurologische Manifestationen bei COVID-19” [20] (20.0%), whereas 6.7% of the respondents reported not using any guideline. Multiple responses were allowed.

The majority of outpatient clinics involved senior physicians (71.2%), followed less frequently by a chief physician (33.3%) or less experienced doctors and therapists (< 51.1%). Most outpatient clinics reported collaboration with other medical fields (88.9%). Regarding professional networking, only 35.6% reported close collaboration with adjacent rehabilitation facilities, while 57.8% exchanged information with nearby ambulant referrers. Patient appointments were mostly self-initiated (51.1%), with referrals from general practitioners (26.7%) being the next common method. Only a small patient number were referred by specialists (11.1%), as in-house referrals from other specialists (6.7%), or other clinics (4.4%). The improvement of ambulatory care represented an important concern for outpatient lead doctors, currently explored in several studies (telemedical support, e.g., “ViCoReK project” “https://www.vicorek-nds.de/”, and “POSCOR Project”, “https://www.poscor.de/”). In an open-response format, survey respondents suggested regular exchanges or meetings with referrers and more time per PCC patient for general practitioners, due to the comprehensive diagnostic assessment required for PCC patients. Some respondents also criticized the insufficient access to information on ambulant PCC specialists and self-help groups. In particular, respondents expressed interest in the establishment of a central directory for outpatient contacts and rehabilitation clinics. At the time of the survey, only 26.7% reported regular organized trainings for outpatient staff.

#### Patient care organization

Based on the self-reports of PCC outpatient doctors, all participating outpatient clinics collectively treated a total of 21,780 patients at least once at the time of this survey, with a mean (SD) of 484 (543) patients per clinic. The majority of PCC follow-up outpatient clinics allocated approximately 30–60 min per patient visit (53.3%). Around two thirds (62.5%) of respondents reported screening patients via questionnaires by mail or phone before physically seeing them. Most clinics saw two patients per day (24.4%). The initial contact between patients and outpatient specialists typically occurred around 29 weeks after acute COVID-19 infection, with a mean (SD) of 28.7 (16.4) weeks. On average, more women (63.3%) were treated than men (36.7%). The most prevalent age group seeking medical advice in outpatient clinics was 40–49 years (51.5%), followed by the age groups of 30–39 years (26.7%) and 50–59 years (20.0%).

#### Patient perspectives

From the perspective of PCC outpatient clinic doctors, the majority of patients seeking medical consultation experience a “strong, 4” (scale from “no distress, 1” to “very strong distress, 5”) degree of symptom-related distress (53.0%). Respondents assumed a “moderate functional impairment, 4” (73.3%, scale from “no impairment, 1” to “severe impairment, 5”) in their clinic population, with pronounced manifestations of fatigue (64.4%), followed by cognitive impairments (15.6%) and dyspnea (13.3%). From the perspective of the lead doctors, patients primarily expected relief from their symptoms (66.7%), reintegration into daily life (60.0%) and to be understood in their complaints (62.2%). Complete cure (37.8%) or exclusion of other somatic causes (37.8%) were considered secondary. On the other hand, lead doctors believed that patient expectations were only partially met (“moderate expectation fulfilment”, 62.5%). The reasons for the incomplete satisfaction of patients from the perspective of PCC outpatient clinic lead doctors were diverse; in an exploratory open-response format, survey participants indicated that the lack of evidence-based therapies contributes to frustration among PCC patients, along with insufficient time and personnel at the treating institution. Additionally, patient-specific factors such as unrealistic expectations, secondary gain from illness, and disease-sustaining comorbidities were mentioned. However, these findings are not intended to be generalized, and the data’s robustness of single opinions is naturally limited.

#### Treatment modalities

**Fig. 3:**
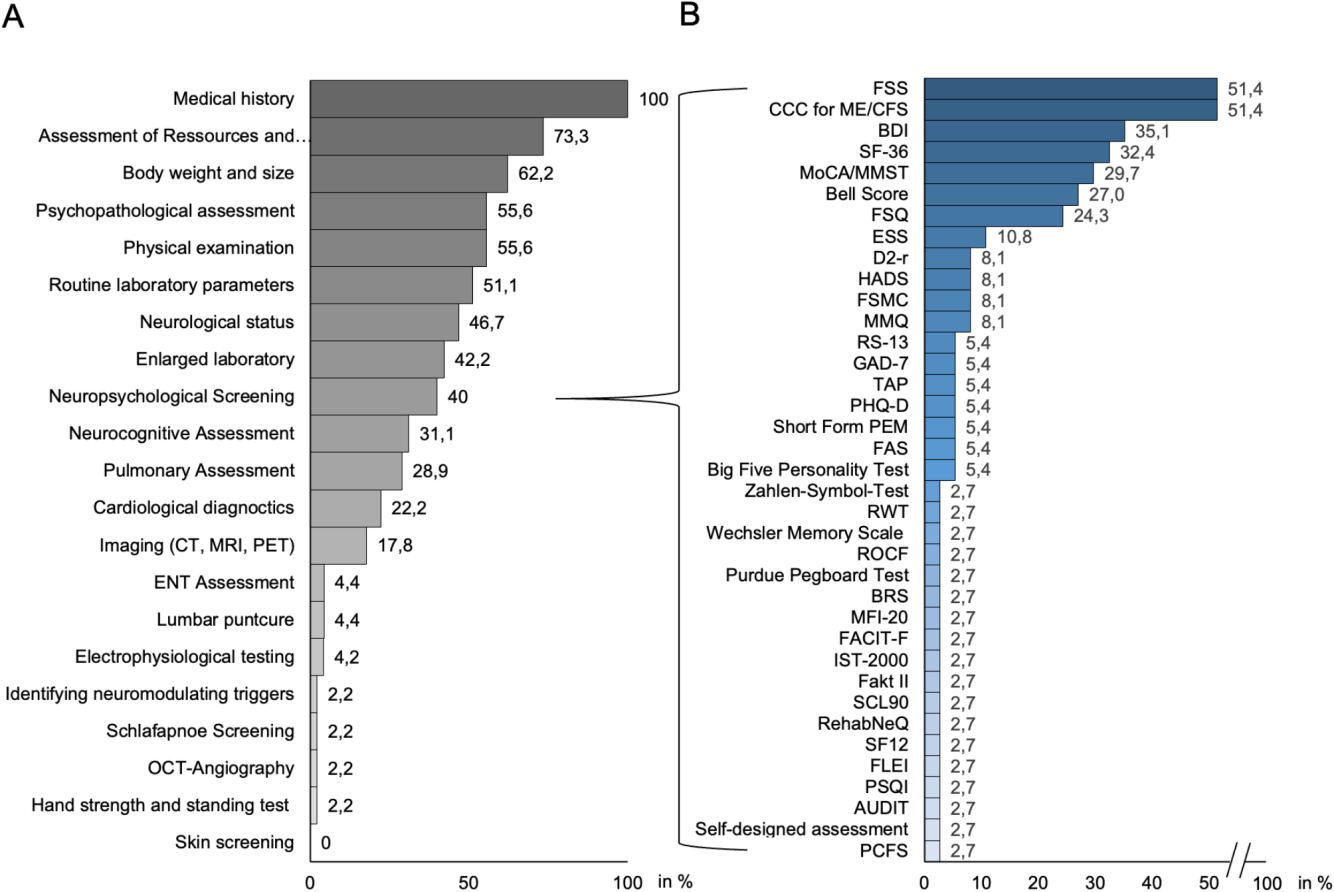
Diagnostic outpatient clinic set-up including the entire offered diagnostic range (**A**) and neurocognitive test diagnostics encompassing five different cognitive domains: Fatigue & Physical Function, Mood & Psychological Symptoms, Memory & Attention, Cognitive Function & Health, Cognitive Performance & Intelligence (**B**). ENT: ear, nose, and throat, OCT-A: optical coherence tomography-angiography. FSS: Fatigue Severity Scale, CCC: Canadian Consensus Criteria, BDI: Beck’s Depression Inventory, SF-36: Short Form-36 Health Survey, MoCa/MMST: Montreal-Cognitive-Assessment-Test/ Mini-Mental-Status-Test, FSQ: Fibromyalgia Survey Questionnaire, ESS: Epworth Sleepiness Scale, HADS: Hospital Anxiety and Depression Scale, FSMC: Fatigue Scale for Motor and Cognitive Functions, MMQ: Multifactorial Memory Questionnaire, RS-13: 13-item Resilience Scale, GAD-7: Generalized Anxiety Disorder 7, TAP: Test of Attentional Performance, PHQ-D: Patient Health Questionnaire, PEM: Post-Exertional Malaise, FAS: Fatigue Assessment Scale, RWT: Regensburger Wortflüssigkeitstest, ROCF: Rey-Osterrieth Complex Figure Test, BRS: Brief Resilience Scale, MFI-20: Multidimensional Fatigue Inventory, FACIT-F: Functional Assessment of Chronic Illness Therapy– Fatigue, IST-2000: Intelligence-Structure-Test, Fakt-II: Frankfurter Adaptiver Konzentrationsleistungs-Test II, SCL-90: Symptom-Checklist, RehabNeQ: Rehabilitation Needs Questionnaire, SF-12: Short Form 12, FLEI: Fragebogen zur geistigen Leistungsfähigkeit, PSQI: Pittsburgh Sleep Quality Index, AUDIT: Alcohol Use Disorders Identification Test, PCFS: Post-COVID-19 Functional Status Scale

Most German outpatient clinics operated within a self-developed structured diagnostic and treatment protocol (73.3%). While 100% of the respondents started with medical history, 73.3% took routine laboratory parameters, and only 55.6% performed a physical examination. Further diagnostic steps differed by medical specialty: neurological status was assessed by 46.7%, psychopathological findings by 55.6%, and further pulmonary assessments were performed by 28.9%. The subsequent diagnostic approach was individually tailored and varied across each consultation (Fig. 3A). Approximately 40% of the consultations regularly utilized a diverse spectrum of cognitive tests (Fig. 3B). The cognitive assessments can be allocated to the following cognitive domains: Fatigue & Physical Function, Mood & Psychological Symptoms, Memory & Attention, Cognitive Function & Health, and Cognitive Performance & Intelligence (Fig. 3B).

**Fig. 4:**
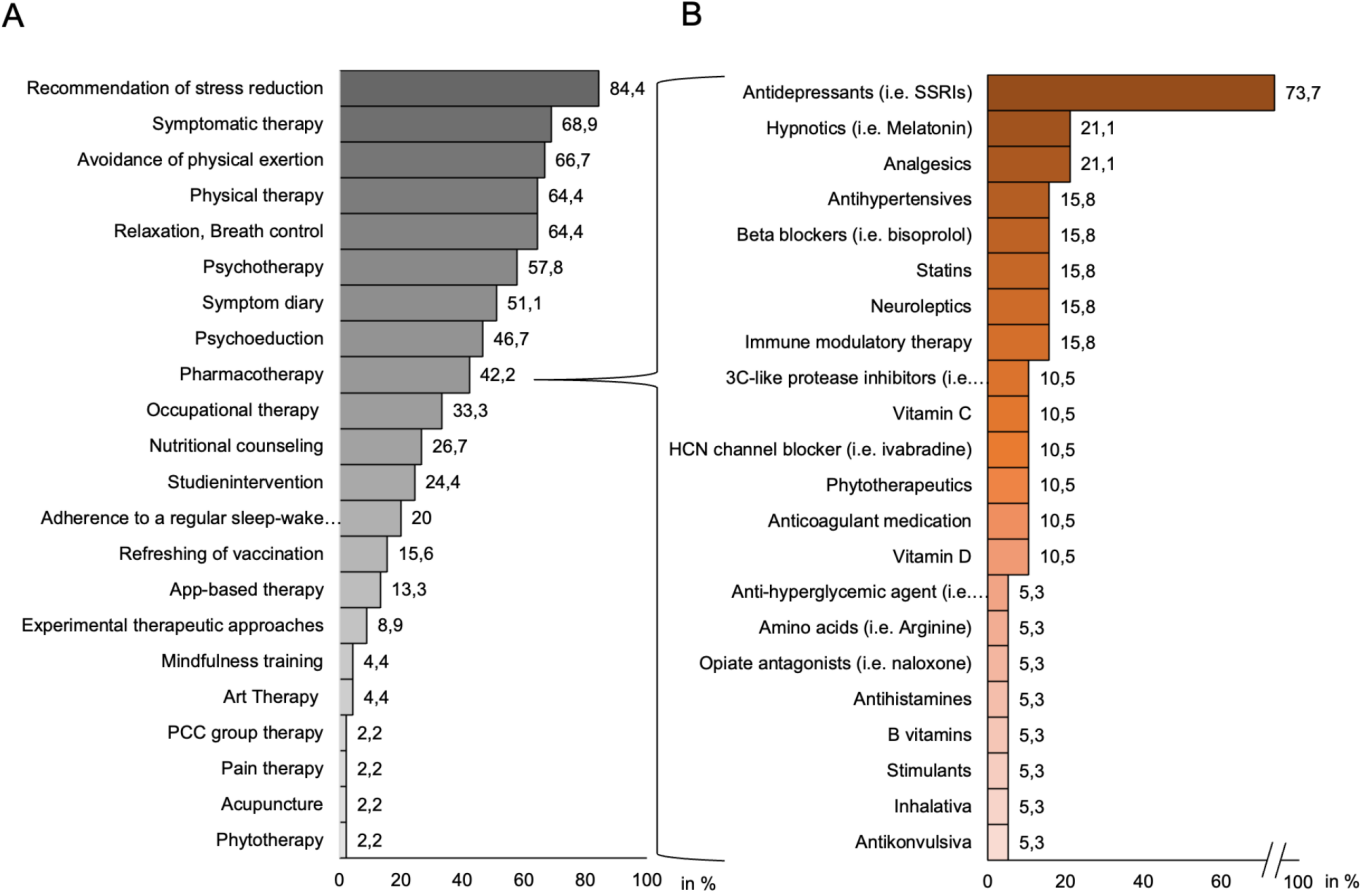
Therapeutic outpatient clinic set-up including the entire offered therapeutic range (**A**) and pharmacotherapy (**B**)

Following diagnostic procedures, approximately 32.3% of patients seen in the outpatient setting received a diagnosis other than PCC. Common differential diagnoses included fatigue of unknown etiology, psychiatric diagnoses such as adjustment disorder and depression, and medical diagnoses such as bronchial asthma of unknown origin and obstructive sleep apnea syndrome. The majority of respondents treated PCC patients symptomatically (68.9%) and guided in stress reduction (84.4%) with additional therapeutic approaches tailored to the specific medical specialization and based on their individual expertise (Fig. 4A). Pharmacotherapy was used by 42.2% of outpatient clinics, whereas only 6.7% of the respondents used immune-modulating medication (Fig. 4B). Experimental approaches such as immune adsorption were applied only by 8.9%; 66.7% respondents said the patients at the follow-up outpatient clinic are screened for clinical studies. Most of the respondents attributed high value to the availability of follow-up outpatient clinics in the care of patients (46.7%), followed by moderate value (35.6%) and very high value (13.3%).

#### Improvement in healthcare structure facilitated by outpatient clinics

Survey responses were summarized in an unstructured and exploratory manner, as follows: demand for more staff, regular training and support for general practitioners, greater exchange among professionals of the field, improved differential diagnosis to identify alternative diagnoses, and enhanced treatment settings (day clinics, group therapy).

#### Comparison between PCC clinics in different medical specialties

Post-hoc comparisons of the characteristics of the two main types of outpatient clinics, namely neurocognitive and respiratory domains, yielded differences in their specific patient cohorts. In the respiratory domain, female patients accounted for 63.9% of the total, while the sex ratio was balanced in the neurocognitive domain (50.2%, *p* < 0.05). Furthermore, significant variations were observed in the main symptoms of patients across outpatient clinic domains (*p* < 0.05). No other characteristics showed statistically significant differences, as detailed in Table 3.

**Table 3:** Perception of different patient cohorts from PCC follow-up outpatient lead Doctors’ perspective

Characteristics	Neurocognitive domain	Respiratory domain	*p*	All outpatient clinics
Number of treated patients (M, SD)	319.6 (276.4)	726.4 (801.9)	n.s.	484 (543.1)
Most treated age group (in y)	40–49	40–49	n.s.	40–49
Sex distribution (♀%)	50.2	63.9	**< 0.05**	60.4
Time to contact after acute infection (in weeks)	26.9	28.1	n.s.	28.7
Patients stress level (1–5)	3.57	3.86	n.s.	3.8
Functional Impairments (1–5)	3.86	3.43	n.s.	3.67
Main symptom				
- Fatigue (%)	64.3	28.6	n.s.	60
- Dyspnea (%)	0	42.9	**< 0.05**	13.3
- Cognitive impairments (%)	14.2	21.4	n.s.	15.6
Different diagnosis (%)	40.7	26.8	n.s.	32.3
Meeting patient expectations (1–5)	3.1	2.9	n.s.	3.0
Perceived significance for patients	3.6	3.4	n.s.	3.7

*PCC* Post-COVID-19 Condition, *M* Median, *SD* Standard Deviation, *n.s.* not significant

## Discussion

We provide a descriptive analysis of German outpatient clinics for PCC utilizing two different methods: (1) identifying and characterizing operating follow-up outpatient clinics through public information and (2) collecting more detailed information by using a survey to outpatient clinic lead doctors to assess their experience in outpatient care of PCC patients. As the demand for PCC follow-up outpatient clinics remains high, new services are continually being established, with a particular focus on meeting the increasing need for neurocognitive care. The distribution of PCC follow-up outpatient clinics shows an imbalance of urban and rural locations. The two identified treatment domains, neurocognitive and respiratory, differ solely in their sex ratio. A significantly higher proportion of females seek assistance for respiratory symptoms, whereas the cohort presenting with neurocognitive symptoms exhibits a balanced sex distribution.

The management of PCC patients remains a significant healthcare challenge as the number of SARS-CoV-2 infections continues to rise, leading to the subsequent emergence of PCC [21]. For economic and medical reasons, the treatment of PCC largely occurs on an outpatient basis, although extended inpatient rehabilitations and custom-tailored care facilities may be feasible in exceptional cases [16, 22]. By January 2024, 112 different outpatient services were identified in Germany. Compared to 2022, both the total number of PCC outpatient clinics, and the proportion of neurocognitive-led clinics increased. Despite variations in methodology and sample representation, Skiba et al. (2024) noted a high demand for consultation appointments and significant utilization of follow-up outpatient clinics as early as the beginning of 2022. Even at that time, fatigue and neurocognitive symptoms were predominantly cited as the leading issues among most PCC patients. The increasing integration of PCC patients into consultations within the neurocognitive domain has since been addressed both medically and organizationally (only in German [23]). Currently employed therapies primarily focus on symptom alleviation and supportive measures depending on the presenting symptoms, reflected by the variety of outpatient clinic types in Germany, where around 40% of all outpatient services cover neurocognitive symptoms, and 31% treat pulmonary symptoms.

Given the limitations in funding and personnel, it is not surprising that public data analysis shows hospital-based PCC outpatient clinics are primarily concentrated in urban areas, particularly those affiliated with university hospitals that support interdisciplinary treatment and research. The advantage of such centers lies in consolidating expertise and experience, as well as facilitating interdisciplinary collaboration [16]. However, this approach requires widespread access to specialized healthcare, which is particularly lacking in rural areas of Germany. These regions were already struggling with significant healthcare distribution challenges, a situation exacerbated by the COVID-19 pandemic [24, 25]. Consequently, severely affected patients may be compelled to undertake significant journeys and endure burdens to receive appropriate treatment. However, mobility poses a significant burden for PCC patients [26]. Moreover, not every clinic addresses every phenotype, i.e., respiratory versus neurological, rendering some clinics unsuitable for certain patients. At this juncture, centralized management or organization could prove beneficial in effectively coordinating clinic offerings and meeting patient demands.

Similar to other countries (e.g. [27]), several guidelines regarding the optimal management of PCC were established in Germany and are largely used as a guide for practice by physicians. The additional attributes regarding staff, consultation hours, and training are congruent with those observed in already established outpatient clinics of other diseases in Germany [28].

The aforementioned diversity of PCC symptoms necessitates expertise of medical specialists to appropriately treat presenting symptoms. Additionally, the prevalence of different PCC symptoms varies according to different studies and at various time points after the initial COVID-19 infection [29]. The highest prevalence among PCC symptoms is fatigue (32%), dyspnea (25%) and cognitive deficits (22%) up to 6 months after acute SARS-CoV-2 infection [30]. This aligns with survey respondents’ perspectives, where fatigue was listed as the leading main symptom, preceding neurocognitive impairments or pulmonary issues. Within the representative survey, outpatient clinics naturally differed in their patients’ main symptoms and sex distribution. However, recent meta-analyses, including one by [31], have identified sex-disaggregated differences in PCC, suggesting sex-specific pathomechanisms and underscoring the necessity for personalized medical treatment. Interestingly, survey respondents perceived high levels of distress in their patients while noticing a significant benefit in the outpatient setting for the treatment of PCC. This underscores the importance of previously established diagnostic instruments and symptom-oriented therapies. PCC patients require personalized treatment regimens; however, the wide array of test instruments and therapeutic approaches compromises the comparability and establishment of medical standards. Moving forward, there is an apparent necessity for a refined understanding of precise diagnostic needs and effective therapies employed, which should be developed. Particularly, a deeper exploration of pathomechanisms is essential, with extensive research already investigating diagnostic biomarkers [12] and (immunomodulatory) therapy approaches [32].

This study has several limitations that need to be addressed. First, data collection relies upon publicly available information, which may not comprehensively capture all existing clinics. To not exceed the scope of the study, only institutionalized PCC outpatient clinics were included in this analysis. This approach, however, overlooks the significant contributions of primary care practitioners in the treatment of PCC. Furthermore, the landscape of outpatient clinics is dynamic; therefore, at the time of publication one must assume at least a slightly altered outpatient landscape in Germany. A centralized and regularly updated overview would mitigate these concerns. The survey results are potentially biased, as they reflect responses from only 40.2% of outpatient clinics that chose to participate, and may not generalize the opinions and experiences of all lead doctors. Furthermore, the setup of outpatient clinics and the evaluation of patient-staff interactions were assessed exclusively from the perspective of the practicing PCC outpatient clinic lead doctors, without accounting for the patient’s viewpoint. Future healthcare analyses should also include the patient perspective.

## Conclusion

Overall, this paper provides a comprehensive resource on the current landscape of outpatient care for PCC patients in Germany. It can serve as the foundation for future improvements in PCC care and promotes further investigation of policy solutions. Regarding the increasing awareness of post-acute infection syndromes (PAIS), the investigated specific infrastructure might be translated to the development of a broader treatment concept in this wide array of diseases. Our findings on the geographical and medical specialty heterogeneity among outpatient clinics can inform future strategies aimed at improving localized and timely access for PCC patients, including telemedical solutions. Standardizing diagnostic and therapeutic methods for PCC treatment can furthermore enhance the quality and effectiveness of care for patients recovering from PCC.

## Supplementary Information

Supplementary Material 1.

## Data Availability

The data supporting the findings of this study are available upon reasonable request from the corresponding author.

## Abbreviations

AUDIT: Alcohol Use Disorders Identification Test
BDI: Beck’s Depression Inventory
BRS: Brief Resilience Scale
CCC: Canadian Consensus Criteria
ENT: Ear-nose-throat
ESS: Epworth Sleepiness Scale
FACIT-F: Functional Assessment of Chronic Illness Therapy– Fatigue
Fakt-II: Frankfurter Adaptiver Konzentrationsleistungs-Test II
FAS: Fatigue Assessment Scale
FLEI: Fragebogen zur geistigen Leistungsfähigkeit
FSMC: Fatigue Scale for Motor and Cognitive Functions
FSQ: Fibromyalgia Survey Questionnaire
FSS: Fatigue Severity Scale
GAD-7: Generalized Anxiety Disorder 7
HADS: Hospital Anxiety and Depression Scale
IST-2000: Intelligence-Structure-Test
ME/CFS: Myalgic encephalomyelitis/chronic fatigue syndrome
MFI-20: Multidimensional Fatigue Inventory
MMQ: Multifactorial Memory Questionnaire
MMST: Mini-Mental-Status-Test
MoCa: Montreal-Cognitive-Assessment-Test
PAIS: Post-acute infection syndrome
PASC: Post-Acute Sequelae of COVID-19
PCS: Post-COVID-19 Syndrome
PCC: Post-COVID-19 Condition
PCFS: Post-COVID-19 Functional Status Scale
PCR: Polymerase chain reaction
PEM: Post-Exertional Malaise
PICS: Post-intensive care syndrome
PQH-D: Patient Health Questionnaire
PSQI: Pittsburgh Sleep Quality Index
RehabNeQ: Rehabilitation Needs Questionnaire
ROCF: Rey-Osterrieth Complex Figure Test
RS-13: 13-item Resilience Scale
SCL-90: Symptom Checklist
SF-12: Short Form-12 Health Survey
SF-36: Short Form-36 Health Survey
TAP: Test of Attentional Performance
