# Supplementary material for: The legacy of the COVID-19 pandemic for the healthcare environment: the establishment of long COVID/ Post-COVID-19 condition follow-up outpatient clinics in Germany
Source: BMC Health Serv Res. 2025 Mar 10;25:360. doi: 10.1186/s12913-025-12521-2 (PMC11895281; doi:10.1186/s12913-025-12521-2)
Supplement: Supplementary file 1 — Supplementary Material 1. [file 12913_2025_12521_MOESM1_ESM.pdf]

PCC follow-up outpatient clinic Assessment

Dear Colleagues,

Thank you for your willingness to participate in this survey about the Post-Covid19 Condition (PCC) follow-up outpatient clinic at your institute. The aim of the survey is to collect information about the currently offered follow-up outpatient clinics and to encourage recommendations for standardizations and improvements in patient care. Your participation is very important. It takes about 5-10 minutes to complete. Please fill out the questions to the best of your knowledge and belief.

Thank you!

Team of the Post-Covid outpatient clinic, Charité Campus Benjamin-Franklin

---

May your answers be evaluated anonymously and possibly published in the course? O Yes O No

Part 1: Formal Structure

1.1 In which catchment area is the Post-Covid19 Condition (PCC) follow-up outpatient clinic of your institute located? Please provide the postal code (ZIP) of the PCC outpatient clinic (e.g., 12203): ___________________________

1.2 Since when has a PCC outpatient clinic been offered at your institute? ___________________________

1.3. Is there a leading medical specialty in which the PCC outpatient clinic is organized?

O Yes
O No

1.3a If yes, which specialties offer a PCC outpatient clinic at your institution?

O Pulmonology
O Cardiology
O Neurology
O Infectiology
O Anesthesiology
O Psychiatry
O Psychosomatics
O Others

If "Other," then which specialty? ___________________________

1.3b If no, which specialties offer a PCC outpatient clinic at your institution?

☐ Pulmonology
☐ Cardiology
☐ Neurology
☐ Infectiology
☐ Anesthesiology
☐ Psychiatry
☐ Psychosomatics
☐ Others

If "Other," then which specialty? ___________________________

1.4 According to which guidelines is the PCC outpatient clinic conducted at your institute?	▢ AWMF S1 Guideline Long/Post-COVID<br>▢ DGN Guideline Neurological Manifestations in COVID-19<br>▢ Empirical Values/Experience<br>▢ Other, already established outpatient clinics, e.g., Chronic Fatigue Syndrome clinic<br>▢ Implementation without guidelines<br>▢ Based on publications<br>▢ Other guidance
Which guidance?	____________________________
1.5 Number and profession of the outpatient clinic staff:	▢ Chief physicians?<br>▢ Senior physicians?<br>▢ Specialists<br>▢ Residents<br>▢ Psychologists or psychotherapists?<br>▢ Nursing staff?<br>▢ Occupational therapists?<br>▢ Physiotherapists?<br>▢ Speech therapists?
Number of involved chief physicians?	____________________________
Number of involved senior physicians?	____________________________
Number of involved specialists?	____________________________
Number of involved residents?	____________________________
Number of involved psychologists or psychotherapists?	____________________________
Number of involved nursing staff?	____________________________
Number of involved occupational therapists?	____________________________
Number of involved physiotherapists?	____________________________
Number of involved speech therapists?	____________________________
1.6 Multidisciplinary networking with other specialties?	O Yes O No
1.7 Is there close exchange/networking with nearby rehabilitation clinics?	O Yes O No
1.8 Is there a close exchange with outpatient follow-up care (general practitioners, specialists)?	O Yes O No
1.9 Possible improvements in PCC outpatient networking	____________________________

1.10 Is there a structured training program for the outpatient staff? O Yes O No

1.11 Who is most likely to refer/admit patients to the PCC outpatient clinic (single selection)?

O Through the patients' own initiative (e.g., via the internet)
O Through general practitioner referral
O Through outpatient specialist referral
O Through referral from other clinics
O Through internal referral from other specialties

1.12 What disorders are treated within the PCC outpatient clinic?

▢ Post/Long-Covid Syndrome
▢ Post-Vacc-Syndrome
▢ Other disorders

What other disorders are treated in the PCC follow-up outpatient clinic?

____

Part 2: Patient Care Organization

2.1 Is there a preliminary screening (by phone, email, in person) with the patients?
O Yes
O No

2.2 Average duration per session per patient:
▢ < 15 minutes
▢ 15-30 minutes
▢ 30-60 minutes
▢ 1-2 hours
▢ > 2 hours

2.3 Average number of sessions per patient per quarter?
▢ 1 x/year
▢ 1 x/quarter
▢ 1 x/month
▢ 1 x/week

2.4 Frequency of consultation offers per week:
▢ On 1 day
▢ On 2 days
▢ On 3 days
▢ On 4 days
▢ On 5 days

2.5 Average number of patients seen per consultation day? ____________________________

2.6 Total number of patients treated so far in your outpatient clinic (approximate indication sufficient): ____________________________

2.7 First contact of the patients after COVID19

infection (average duration in weeks, approximate indication sufficient): ____________________

2.8 Did all patients have a confirmed COVID19 infection by PCR or antigen test?
O Yes
O No

2.9 The approximate gender distribution of the patients in the PCC outpatient clinic?
Female — — — — — — — — — — — Male
Please mark the ratio on the scale

2.10 The average age of the patients in the PCC follow-up outpatient clinic?
O 18-29 years
O 30-39 years
O 40-49 years
O 50-59 years
O 60-69 years
O 70-79 years
O ≥ 80 years

Part 3: Patient Perspectives

3.1 How subjectively burdened are your patients on average at the initial presentation?
O Not at all
O Slightly
O Moderately
O Strongly
O Very Strongly

3.2 How do you estimate the average functional level of the presenting patients?

O No functional impairment (fully capable in work and private life)
O Negligible functional impairment (subjectively burdened, but no restriction in carrying out activities)
O Mild functional impairment (professional and private activities require effort but are feasible)
O Moderate functional impairment (restriction of professional and private activities)
O Severe functional impairment (independent care no longer possible)

3.3 Main symptom of the majority of patients at initial presentation (single selection)?
O Fatigue
O Pulmonary symptoms (e.g., dyspnea, shortness of breath)
O cardiovascular symptoms (e.g., tachycardia, palpitations, blood pressure fluctuations)
O Dizziness
O Taste and smell disorders
O Headaches
O Concentration and memory problems
O Problems in the musculoskeletal system (muscle, bone pain)
O Reduced performance
O Other symptoms

3.4 Other frequently mentioned burdensome symptoms (multiple selection possible):
▢ Fatigue
▢ Pulmonary symptoms (e.g., dyspnea, shortness of breath)
▢ cardiovascular symptoms (e.g., tachycardia, palpitations, blood pressure fluctuations)
▢ Dizziness

	▢ Taste and smell disorders<br>▢ Headaches<br>▢ Concentration and memory problems<br>▢ Problems in the musculoskeletal system (muscle, bone pain)<br>▢ Reduced performance<br>▢ Other symptoms
3.5 What do you think are the primary expectations of your patients (multiple selections possible)?	▢ “Healing“, complete recovery<br>▢ Symptom reduction<br>▢ Re-engagement in daily life/work<br>▢ Feeling heard/understood<br>▢ Exclusion of somatic causes<br>▢ Getting a diagnosis they can live with<br>▢ Other expectations
If "Other expectation," then:	___________________________
3.6 Are these expectations met in your estimation?	O Not at all<br>O Slightly<br>O Moderately<br>O Strongly<br>O Completely
3.7 If the expectations are not fully met, what do you think is the main reason?<br>Please briefly enter free text.	___________________________

Part 4: Treatment modalities

4.1 Is there a fixed structured diagnostic program that all patients go through in follow-up outpatient clinic?	O Yes<br>O No
4.2 Which diagnostics are an essential part of the outpatient clinic program?<br>Multiple selections possible:	▢ Medical history<br>▢ Physical internal examination<br>▢ Neurological status<br>▢ Psychopathological findings or psychiatric history<br>▢ Dermatological screening<br>▢ ENT assessment, especially smell testing<br>▢ Survey of psychosocial changes, occupation, partnership, resources, stressors<br>▢ Survey of weight, BMI, weight dynamics<br>▢ Neurocognitive testing<br>▢ Psychological/psychotherapeutic screening<br>▢ Cardiological functional diagnostics (TTE, ECG)<br>▢ Pulmonary functional diagnostics (PFT, spirometry)<br>▢ Routine laboratory (CBC, infection, creatinine, AST/ALT/GT, bilirubin)

	▢ Extended laboratory (rheumatology, CK, antibodies, etc.)<br>▢ Lumbar puncture (LP)<br>▢ Electrophysiology<br>▢ Imaging (CT, MRI, PET)<br>▢ Other diagnostics not listed here
If other diagnostics, then which?	___________________________
If LP, which markers are examined? (Cell count, specific antibodies, etc.)	___________________________
4.3 Which neurocognitive tests/self-assessment tools are routinely performed?	▢ Beck's Depression Inventory (BDI)<br>▢ Multifactorial Memory Questionnaire (MMQ)<br>▢ Canadian Consensus Criteria for CFS/ME<br>▢ Scale of the degree of disability according to David Bell (Bell Score)<br>▢ Fatigue severity scale (FSS)<br>▢ Chalder fatigue scale (CFQ)<br>▢ Montreal-Cognitive-Assessment-Test (MoCA)/ Mini-Mental-State-Test (MMST)<br>▢ Short Form Health Survey (SF-36) to assess health-related quality of life<br>▢ Other tests/questionnaires
If “other tests“, then:	___________________________
4.4 What percentage of presenting patients do not receive the diagnosis "Post-Covid19 Condition" after completing the diagnostics?	0 50 100<br>— — — — — — — — — — —<br>*Please mark the ratio on the scale*
4.5 If "Post-Covid19 Condition" is not the final diagnosis, what are the 3 most common alternative differential diagnoses?	___________________________

4.6 The following therapy options/interventions are offered and carried out:

▢ Symptom diary
▢ Symptomatic therapy/referral to specialty
▢ Recommendations to avoid stress
▢ Avoidance of excessive physical exertion
▢ Instruction for relaxation exercises, breathing exercises
▢ Training of circadian sleep-wake rhythm
▢ Psychoeducation
▢ Occupational therapy
▢ Physiotherapy
▢ Psychotherapy (CBT, TP)
▢ Nutritional counseling
▢ Telemedicine or app-based consultation (e.g., MagicApp)
▢ Recommendation for re-vaccination

▢ Pharmacotherapy
▢ Immunomodulatory pharmacotherapy
▢ Experimental therapy approaches (e.g., immunoadsorption, lipid apheresis, hyperbaric intervention in studies)
▢ Other therapy

If "other therapy", then:	___________________________
If pharmacotherapy, which groups of medications (antidepressants, immunosuppressants, analgesics, etc.):	___________________________
If immunomodulatory pharmacotherapy, then:	___________________________
4.7 What type of rehabilitation is offered to PCC patients?	___________________________
4.8 Are patients screened and/or recruited for possible study participation in the PCC follow-up outpatient clinic?	O Yes<br>O No
4.9 How do you assess the importance/benefit of the Post-Covid19 follow-up outpatient clinic for your patients' coping with the disease?	▢ Not at all<br>▢ Slightly<br>▢ Moderately<br>▢ high usefulness<br>▢ very high usefulness

Part 5: Improvement in healthcare structure facilitated by outpatient clinics

5.1 Possible improvements in the care structure for PCC patients through the Post-Covid19 follow-up outpatient clinic? Please enter free text	___________________________

PCS Sprechstunde Assessment

Sehr geehrte Kolleginnen und Kollegen,

vielen Dank für Ihre Bereitschaft, an dieser Umfrage über die Post-Covid-Syndrom (PCS) Sprechstunde an Ihrem Institut teilzunehmen. Ziel der Umfrage ist es, Informationen über die aktuell angebotenen Sprechstunden zu sammeln und Empfehlungen für Vereinheitlichungen und Verbesserungen in der Patientenversorgung anzuregen. Ihre Teilnahme ist dabei sehr wichtig. Die Bearbeitung dauert ca. 5-10 Minuten. Bitte füllen Sie die Fragen nach bestem Wissen und Gewissen aus.

Vielen Dank!

Team der Post-Covid Sprechstunde, Charité Campus Benjamin-Franklin

---

Dürfen Ihre Antworten anonym ausgewertet und ggf. im Verlauf publiziert werden? ◯ Ja ◯ Nein

Teil 1: Formaler Rahmen

1.1 In welchem Einzugsgebiet wird die Post-Covid Syndrom (PCS) Sprechstunde an Ihrem Institut angeboten? Bitte Postleitzahl (PLZ) der Sprechstunde angeben (z.B. 12203): ____________________

1.2 Seit wann wird an Ihrem Institut eine PCS Sprechstunde angeboten? ____________________

1.3. Gibt es eine federführende medizinische Fachrichtung, in der die PCS Sprechstunde organisiert wird?

◯ Ja
◯ Nein

1.3a Falls ja, welche Fachrichtungen bieten an Ihrer Institution eine PCS Sprechstunde an?

◯ Pulmologie
◯ Kardiologie
◯ Neurologie
◯ Infektiologie
◯ Anästhesie (Schmerz, Palliativmedizin)
◯ Psychiatrie
◯ Psychosomatik
◯ Andere

Wenn "Andere", dann welche Fachrichtung? ____________________

1.3b Falls nein, welche Fachrichtungen bieten an ihrer Institution eine PCS Sprechstunde an?

☐ Pulmologie
☐ Kardiologie
☐ Neurologie
☐ Infektiologie
☐ Anästhesie (Schmerz, Palliativmedizin)
☐ Psychiatrie
☐ Psychosomatik
☐ Andere

Wenn "Andere", dann welche Fachrichtung? ____________________

1.4Nach welchem Leitfaden wird die PCS Sprechstunde in Ihrem Institut durchgeführt?	☐ AWMF S1-Leitlinie Long/ Post-COVID<br>☐ DGN-Leitlinie Neurologische Manifestationen bei COVID-19<br>☐ Erfahrungswerte<br>☐ Andere, bereits etablierte Sprechstunden, z.B. Chronic Fatigue Syndrom Sprechstunde<br>☐ Durchführung ohne Leitfaden<br>☐ anhand von Publikationen<br>☐ andere Orientierungshilfe
Welche Orientierungshilfe?	__________________________________
1.5 Anzahl und Profession der Sprechstunden-Mitarbeitenden	☐ Chefärzt:innen<br>☐ Oberärzt:innen<br>☐ Fachärzt:innen<br>☐ Ärzt:innen<br>☐ Psychologen und Psychotherapeutinnen<br>☐ Pflegepersonal<br>☐ Ergotherapie<br>☐ Physiotherapie<br>☐ Logopädie<br>☐ Andere
Anzahl der beteiligten Chefärzt:innen?	____________________________
Anzahl der beteiligten Oberärzt:innen?	____________________________
Anzahl der beteiligten Fachärzt:innen?	____________________________
Anzahl der beteiligten Ärzt:innen?	____________________________
Anzahl der beteiligten Psychologen oder Psychotherapeutinnen?	____________________________
Anzahl des beteiligten Pflegepersonals?	____________________________
Anzahl der beteiligten Ergotherapeut:innen?	____________________________
Anzahl der beteiligten Physiotherapeut:innen?	____________________________
Anzahl der beteiligten Logopäd:innen?	____________________________
1.6 Multidisziplinäre Vernetzung mit anderen Fachdisziplinen?	◯ Ja ◯ Nein
1.7 Besteht ein enger Austausch/ Vernetzung mit umliegenden Rehakliniken?	◯ Ja ◯ Nein

1.8 Besteht ein enger Austausch mit der ambulanten Weiterversorgung (Hausärzt:innen, Fachärzt:innen)?

◯ Ja ◯ Nein

1.9 Mögliche Verbesserungen der ambulanten Vernetzung

____

1.10 Besteht ein strukturiertes Weiterbildungsangebot für das Personal der Sprechstunde?

◯ Ja ◯ Nein

1.11 Über wen erfolgt am ehesten die Überweisung/Aufnahme der Patient:innen in die Post-Covid-Sprechstunde (Einfachnennung)?

◯ durch Eigeninitiative der Pat. (z.B. über Internet)
◯ durch amb. hausärztliche Zuweisung
◯ durch amb. fachärztliche Zuweisung
◯ durch Überweisung aus anderen Kliniken
◯ durch interne Überweisung aus anderen Fachrichtungen

1.12 Welche Störungsbilder werden im Rahmen der PCS Sprechstunde behandelt?

☐ Post/Long-Covid-Syndrom
☐ Post-Vacc-Syndrom
☐ noch weitere Störungsbilder

Welche weiteren Störungsbilder werden in PCS Sprechstunde behandelt?

____

Teil 2: Patientenversorgung

2.1 Findet ein Vorgespräch (über Telefon, eMail, persönlich) mit den Patient:innen statt?

◯ Ja
◯ Nein

	< 15 Minuten	15-30 Minuten	30-60 Minuten	1-2 Stunden	> 2 Stunden
2.2 Durchschnittliche Dauer pro Sitzung pro Patient insgesamt:	☐	☐	☐	☐	☐

2.3 Durchschnittliche Anzahl an Sitzungen pro Patient und pro Quartal?

☐ 1 x/ Jahr
☐ 1 x/ Quartal
☐ 1x/ Monat
☐ 1x/ Woche

2.4 Häufigkeit des Sprechstundenagebots pro Woche:

☐ an 1 Tag
☐ an 2 Tagen
☐ an 3 Tagen
☐ an 4 Tagen
☐ an 5 Tagen

2.5 Durchschnittliche Anzahl der gesehenen Patient:innen pro Sprechstundentag?

____

2.6 Gesamtzahl aller bisher behandelten Patient:innen in Ihrer Sprechstunde (ungefähre Angabe ausreichend):

____

2.7 Erste Kontaktaufnahme der Patient:innen nach COVID-Infektion (Durchschnittliche Dauer in Wochen, ungefähre Angabe ausreichend):

____

2.8 Bestand bei allen Patient:innen eine gesicherte COVID-Infektion mittels PCR oder Ag-Test?:

- ◯ Ja
- ◯ Nein

2.9 Die ungefähre Geschlechterverteilung der Patient:innen der Post-Covid-Sprechstunde?

weiblich ———————————— männlich

Setzen Sie auf der obrigen Skala eine Markierung

2.10 Das durchschnittliche Alter der Patient:innen der Sprechstunde?

- ◯ 18-29 Jahre
- ◯ 30-39 Jahre
- ◯ 40-49 Jahre
- ◯ 50-59 Jahre
- ◯ 60-69 Jahre
- ◯ 70-79 Jahre
- ◯ ≥ 80 Jahre

Teil 3: Patientenperspektive

3.1 Wie sind Ihre Patient:innen bei Erstvorstellung durchschnittlich subjektiv belastet?

- ◯ gar nicht
- ◯ wenig
- ◯ mittelstark
- ◯ stark
- ◯ sehr stark

3.2 Wie schätzen Sie das durchschnittliche Funktionsniveau der sich vorstellenden Patient:innen ein?

- ◯ keine funktionelle Einschränkung (vollständige Leistungsfähigkeit in Berufs- und Privatleben)
- ◯ vernachlässigbare funktionelle Einschränkung (subj. belastet, jedoch keine Einschränkung in der Durchführung von Aktivitäten)
- ◯ leichte funktionelle Einschränkung (berufliche und private Aktiväten erfordern Anstrengung, sind aber durchführbar)
- ◯ mäßige funktionelle Einschränkung (einschränkung von beruflichen und privaten Aktivitäten)
- ◯ schwere funktionelle Einschränkung (selbstständige Versorgung nicht mehr möglich)

3.3 Hauptsymptom der mehrheitlichen Patient:innen bei Erstvorstellung (Einfachnennung)?

- ◯ Tagesmüdigkeit
- ◯ Pulmonale Symptome (i.e. Dyspnoe, Kurzatmigkeit)
- ◯ Kreislaufbeschwerden (i.e. Tachykardie, "Herzstolpern", Blutdruckschwankungen)
- ◯ Schwindel
- ◯ Geschmack- und Geruchsstörungen
- ◯ Kopfschmerzen
- ◯ Konzentrations- und Gedächtnisstörungen
- ◯ Probleme im muskoloskeletalen System (Muskel-, Knochenschmerzen)
- ◯ Belastungsminderung
- ◯ Andere Symptome

(Hauptgrund der Vorstellung)

Wenn "Andere Symptome", dann

____

3.4 Weitere, häufig genannte belastende Symptome (Mehrfachnennung möglich):	☐ Tagesmüdigkeit<br>☐ Pulmonale Symptome (i.e. Dyspnoe, Kurzatmigkeit)<br>☐ Kreislaufbeschwerden (i.e. Tachykardie, "Herzstolpern", Blutdruckschwankungen)<br>☐ Schwindel<br>☐ Geschmacks- und Geruchsstörungen<br>☐ Kopfschmerzen<br>☐ Konzentrations- und Gedächtnisstörungen<br>☐ Probleme im muskoloskeletalen System (Muskel-, Knochenschmerzen)<br>☐ Belastungsminderung<br>☐ Andere Symptome
3.5 Was sind Ihrer Ansicht nach die primären Erwartungen Ihrer Patienten (Mehrfachnennung möglich)?	☐ "Heilung", vollständige Genesung<br>☐ Reduktion der Symptome<br>☐ Wiederteilhabe am Alltag/ Berufsleben<br>☐ "Gehört"/ Verstanden werden<br>☐ Ausschluss somatische Ursache<br>☐ Eine Diagnose bekommen, mit der die Patienten leben können<br>☐ Andere Erwartung
Wenn "Andere Erwartung", dann:	__________________________________
3.6 Werden diese Erwartungen Ihrer Einschätzung nach erfüllt?	○ gar nicht<br>○ wenig<br>○ mittel<br>○ stark<br>○ vollständig
3.7 Falls die Erwartungen nicht vollständig erfüllt werden, woran liegt das Ihrer Meinung nach hauptsächlich? Bitte kurz Freitext eingeben.	__________________________________

Teil 4: Inhalte der Versorgung

4.1. Gibt es ein fest strukturiertes diagnostisches Programm, das von allen Patienten in der Sprechstunde durchlaufen wird?	○ Ja<br>○ Nein

4.2 Welche Diagnostik ist essentieller Bestandteil der Patientenvorstellung? Mehrfachnennung möglich	☐ Krankheitsanamnese<br>☐ Körperliche internistische Untersuchung durch geschultes Fachpersonal<br>☐ Neurologischer Status<br>☐ Psychopathologischer Befund bzw. psychiatrische Anamnese<br>☐ Dermatologisches Screening<br>☐ HNO-Einschätzung, insb. Riechtestung<br>☐ Erhebung psychosozialer Veränderungen, Beruf, Partnerschaft, Ressourcen, Stressoren<br>☐ Erhebung von Gewicht, BMI, Gewichtsdynamik<br>☐ Neurokognitive Testung<br>☐ Psychologisches/ psychotherapeutisches Screening<br>☐ Kardiologische Funktionsdiagnostik (TTE, EKG)<br>☐ Pulmonale Funktionsdiagnostik (LuFo, Spiro)<br>☐ Routine Labor (BB, Infekt, Krea, AST/ALT/GT, Bilirubin)<br>☐ Erweitertes Labor (Rheuma, CK, Antikörper, etc.)<br>☐ Lumbalpunktion (LP)<br>☐ Elektrophysiologie<br>☐ Bildgebung (cCT, cMRT, PET)<br>☐ Andere, hier nicht genannte Diagnostik
Wenn Andere Diagnostik, dann welche?	______________________________
Wenn LP, welche Marker werden untersucht? (Zellzahl, bestimmte Antikörper, etc.)	______________________________
4.3Welche neurokognitiven Tests/ Selbstauskunftsinstrumente werden standardmäßig durchgeführt?	☐ keine<br>☐ Beck's Depression Iventar (BDI)<br>☐ Multifactorial Memory Questionnaire (MMQ)<br>☐ Canadian Consensus Kriterien für CFS/ME<br>☐ Skala des Grads der Behinderung nach David Bell (Bell Score)<br>☐ Fatigue severity scale (FSS)<br>☐ Chalder fatigue scale (CFQ)<br>☐ Montreal-Cognitive-Assessment-Test (MoCA)/ Mini-Mental-State-Test (MMST)<br>☐ Short Form Gesundheitsfragebogen (SF-36) zur Erhebung der gesundheitsbezogenen Lebensqualität<br>☐ Andere Tests/Fragebögen:
Wenn "Andere Tests", dann:	______________________________
4.4 Zu wieviel % erhalten die sich vorstellenden Pat. nicht die Diagnose "Post-Covid-Syndrom" nach Abschluss der Diagnostik?	0 50 100<br>*Setzen Sie auf der obrigen Skala eine Markierung*

4.5 Sofern "Post-Covid-Syndrom" nicht die abschließende Diagnose ist, was sind die 3 häufigsten alternativen Differentialdiagnosen?

4.6 Folgende Therapieoptionen/Interventionen werden angeboten und durchgeführt:

- ☐ Symptomtagebuch
- ☐ Symptomatische Therapie/Überweisung an Fachrichtung
- ☐ Empfehlungen zur Stressvermeidung
- ☐ Vermeidung zu starker körperlicher Anstrengung
- ☐ Anleitung für Entspannungsübungen, Atemübungen
- ☐ Training der circadianen Schlaf-Wach-Rhythmik
- ☐ Psychoedukation
- ☐ Ergotherapie
- ☐ Physiotherapie
- ☐ Psychotherapie (VT, TP)
- ☐ Ernährungsberatung
- ☐ Telemedizin- oder App-gestützte Beratung (i.e. MagicApp)
- ☐ Empfehlung zur erneuten Impfung
- ☐ Pharmakotherapie
- ☐ Immunmodulatorische Pharmakotherapie
- ☐ Experimentelle Therapieansätze (z.B. Immunabsorption, Lipidapherese, hyperbare
- ☐ Intervention im Rahmen von Studien
- ☐ Andere Therapie

Wenn andere Therapie, dann: ______________________________

Wenn Pharmakotherapie, dann welche Gruppen von Medikamenten (Antidepressiva, Immunsuppressiva, Analgetika, etc.): ______________________________

Wenn immunmodulierende Pharmakotherapie, dann: ______________________________

4.7 Welche Art von Rehabilitation wird den PCS Patient:innen angeboten? ______________________________

4.8 Werden Patint:innen in der PCS Sprechstunde für mögliche Studienteilnahmen gescreent und/oder rekrutiert?

- ◯ Ja
- ◯ Nein

4.9 Wie schätzen Sie den Stellenwert/ Nutzen der Post-Covid-Sprechstunde für die Krankheitsbewältigung Ihrer Patient:innen ein?

- ☐ gar nicht
- ☐ leicht
- ☐ mittel
- ☐ hoher Nutzen
- ☐ sehr hoher Nutzen

Teil 5: Verbesserungen der Versorgungsstruktur durch die PCS Sprechstunde

5.1 Mögliche Verbesserungen der Versorgungsstruktur der PCS Patient:innen mittels Post-Covid-Sprechstunde? Bitte Freitext eingeben ______________________________
